# Fibrous hamartoma of infancy: radiologic features and literature review

**DOI:** 10.1186/s12891-019-2743-5

**Published:** 2019-08-03

**Authors:** Yang Ji, Peizhen Hu, Chuanshan Zhang, Qingguo Yan, Hong Cheng, Ming Han, Zhe Huang, Xia Wang, Heng Li, Yuedong Han

**Affiliations:** 1 Department of Imaging Center, First Affiliated Hospital, Xi’an Medical University, Xi’an, ShaanXi China; 2 Department of Pathology, First affiliated Hospital of Air Force Medical University, Xi’an, ShaanXi China; 3 Department of Pathology, Third Central Hospital, Tianjin, China; 4 Department of Radiology, GaoXin Hospital, Xi’an Jiao Tong University, No.16, South Tuanjie Road, Xi’an, ShaanXi China

**Keywords:** Fibrous hamartoma of infancy, Soft tissue, Radiologic imaging

## Abstract

**Background:**

Fibrous hamartoma of infancy(FHI) is a rare benign lesion most frequently occurring within the first year of life. So far, just over 200 cases have been reported in the English literature, in which the radiologic findings of FHI have not been fully described. Herein, 2 adult cases of FHI receiving treatment in our hospital and the published cases searched on PubMed are reviewed, with the emphasis on the discussion of the spectrum of MR findings and their histologic correlation.

**Case presentation:**

We present two adult cases who aged 47 years and 19 years with slow growing masses beginning from their childhood in the posterior craniocervical area. On CT and MR imaging, the tumours showed as the superficially located lesions with ill-defined margins that involved the subcutaneous layer and its underlying muscles. The size of the lesions were 21.3 × 16.7 × 16 cm in case 1 and 20.2 × 19.3 × 13.6 cm in case 2. The tumours demonstrated heterogeneous intensities/signals with the adipose tissue presenting as the disperse strands or small focus of fatty intensity/signal. Parallel or whirling appearance, and dilated vessels were delineated in the cases. Contrast enhancement was administered in case 1 and marked enhancement was found.

**Conclusions:**

The usually observed manifestation of FHI on CT and/or MR imaging is the strands of adipose/fibrous intensities traversing the lesions, with the characteristic parallel or whirling appearance in some cases. The tumours with ill-defined margins have the tendency to involve the underlying muscles. Some fibroblastic and adipocytic tumours should be ruled out in differential diagnosis.

## Background

Fibrous hamartoma of infancy(FHI) is a rare benign lesion. It was first described by Reye in 1956 and was named as FHI by Enzinger in 1965 [1, 2]. The vast majority of the cases (91%) occur within the first year of life and about 25% of them are congenital [2]. The lesions most frequently involve the superficial soft tissues and may occur anywhere in the body, for example, axilla, upper arm, trunk, external genital area, and so on. The lesions’ histological appearance of the three different tissues: fibrous trabeculae, mature adipose cells, and immature mesenchymal tissues, is specific for diagnosis of FHI [1–4].

Up to now, just over 200 cases of FHI have been reported [2–6]. However, no images of series of the lesions have been sufficiently investigated and published. Although 5 cases of FHI were included in a case series reported by Lee et al. [7], no detailed radiologic demonstrations were described. On MR imaging, there were reports that the tumours of FHI had signal intensity similar to fibromatosis, and there were also reports that the tumours of FHI had signal intensity similar to lipoma [8–10]. Therefore, the relationship between the different MR imaging appearances and their corresponding histologic components of FHI should be further investigated. In addition, no radiologic findings of FHI in adults have been reported as yet. Herein, we described 2 cases of FHI in adults beginning from their childhood. The radiologic manifestations of the 2 cases, especially the MR imaging findings, together with the reported cases in the English literature, were described.

## Case presentation

### Case 1

**Fig. 1:**
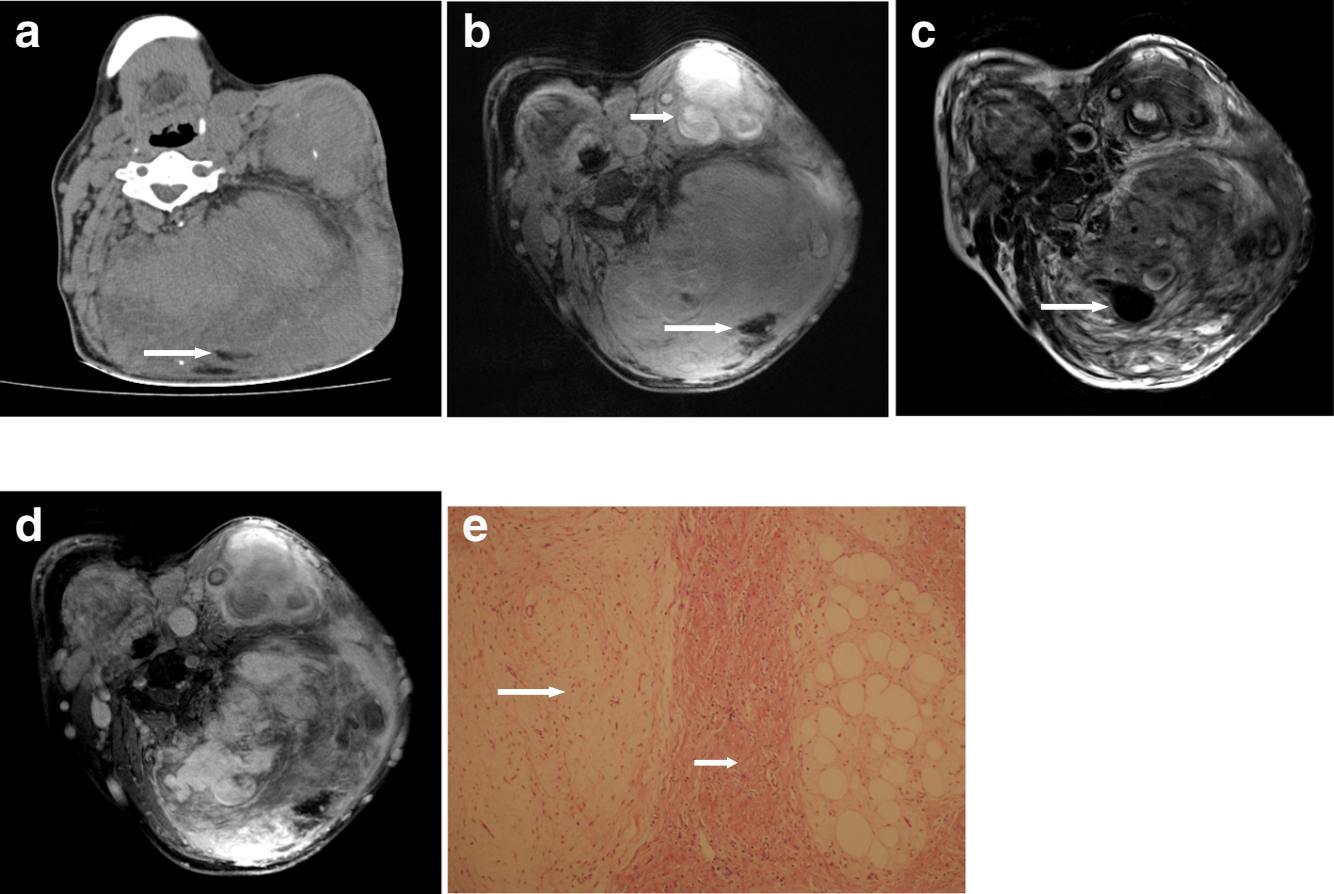
**a** Noncontrast CT scan shows stripes of adipose in the mass (arrow) with speckled calcifications. **b** Axial fat suppressed T_1_-weighted MR image delineates hypointensity of adipose (arrow) and hyperintensity of multifocal subacute hemorrhages (short arrow). **c** Axial T_2_-weighted MR image shows the whirling appearance in the lobules combined with the signal void of vessels (arrow). **d** Contrast-enhanced axial fat suppressed T_1_-weighted MR image demonstrates heterogeneous enhancement of the mass. **e** Photomicrograph of pathologic specimen shows an admixture of mature adipose tissue, nodular aggregate of immature mesenchyme(arrow), and dense bundle of fibrocollagenous tissue (short arrow) (H&E,× 200)

An adult male, age 47 years (566 months), presented with a 42 years (505 months) history of a slow growing mass in the left posterior craniocervical area measuring 21.3 × 16.7 × 16 cm with multiple dilated vessels on its surface. The patient underwent operation at the age of 26 in another institution, but the surgery had to be terminated at the very beginning because of severe bleeding. Radiography showed a huge soft tissue mass with faint punctuate calcifications. On nonenhanced CT scan, the lesion presented as a multi-lobular heterogeneous density mass at the subcutaneous tissue with mixed low- and high attenuations suggestive of adipose and calcifications (Fig. 1a). No boundaries were seen between the mass and the surrounding structures with the latter being markedly displaced. MR imaging reasserted the demonstrations above except for calcifications, and further showed whirling appearance in the lobules and multiple tortuous signal voids suggesting dilated tumour vessels with the largest diameter of 23.2 mm both on fat-supressed T1WI and T2WI (Fig. 1b,Fig. 1c). In addition, foci of heterogeneous hyperintensity were also demonstrated, suggestive of subacute hemorrhage. The mass was heterogeneously enhanced after the intravenous administration of Gd-DTPA(0.1 mmol/kg) (Fig. 1d). The patient underwent subtotal excision of the tumour which was 3100 g in weight and recovered from surgery. Histopathology disclosed an FHI with rich collagenous tissue, islands of adipose, and nests of immature mesenchyme (Fig. 1e).

### Case 2

**Fig. 2:**
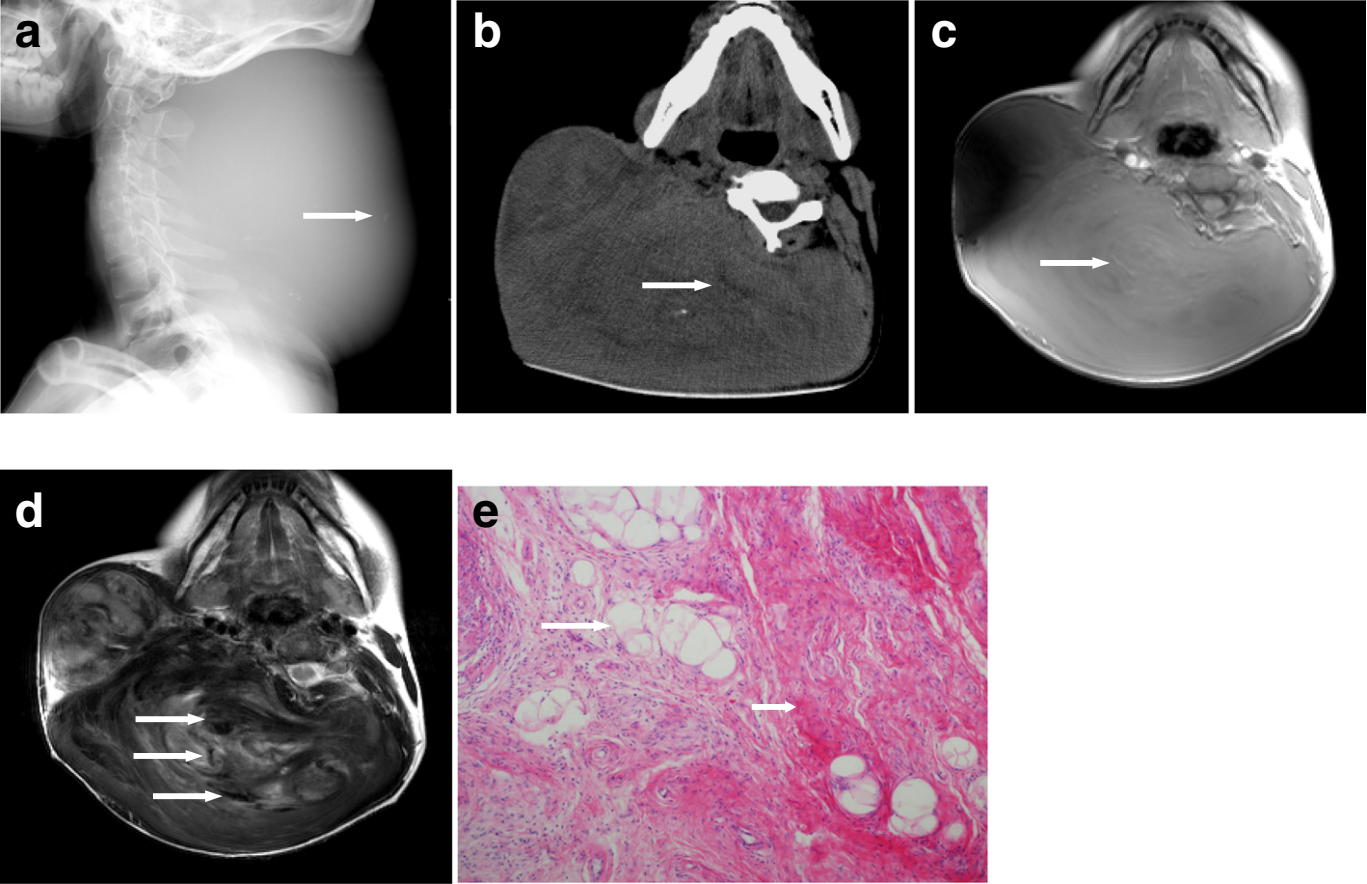
**a** Lateral radiograph of the craniocervical area shows a giant soft-tissue mass with faint punctuate calcifications (arrow) and obvious compression of the skull. **b** Noncontrast CT scan shows low intensity of adipose (arrow) and high intensity of mineralization in the lobular mass. **c** Axial T_1_-weighted MR image demonstrates fine strands of high signal adipose (arrow). **d** Axial T_2_-weighted MR image delineates the whirling appearance in the lobules and multiple serpiginous signal voids (arrows). **e** Photomicrograph of pathologic specimen shows an admixture of islands of mature adipose tissue(arrow), scattered immature mesenchymal cells, and dense bundles of fibrous tissue (short arrow) (H&E,× 200)

An adult female, age 19 years (232 months), presented with a painless, right sided posterolateral craniocervical mass measuring 20.2 × 19.3 × 13.6 cm from her birth as a neonate. She did not undergo any medical treatment until she was referred to our institution. Radiography showed a huge soft tissue mass with faint punctuate calcifications. The underlying structures were compressed obviously (Fig. 2a). Nonenhanced CT scan demonstrated a multi-lobular ill-circumscribed heterogeneous density mass locating at the subcutaneous layer with mixed low and high attenuations indicative of adipose and calcifications (Fig. 2b). MR imaging confirmed the demonstrations above except for calcifications, and further delineated whirling appearance in the lobules and focal tortuous signal voids suggesting dilated tumour vessels (Fig. 2c,Fig. 2d). The patient underwent complete resection of the tumour which was 2750 g in weight and fully recovered from surgery. The histologic examination was indicative of FHI with rich collagenous tissue, interspersed adipose, and scattered immature mesenchymal cells (Fig. 2e).

## Discussion

FHI is a distinctly rare lesion occasionally reported in the English literature [1–4]. Only over 200 cases were reported [2, 4, 6, 11] in literature reviews of FHI with enough clinical and pathologic delineations. FHIs are almost all benign masses with the exception of 2 cases in which sarcoma occurred [11]. Although FHIs may present at any time from birth to middle age, they almost always occur in the first two years of life with boys more frequently involved than girls (2.4:1) [2–5]. Ordinarily, the tumours present as a solitary nontender mass in the subcutaneous soft tissue with the most common locations being axillary regions, trunk, upper arms, chest wall, neck, and external genital areas [1–4]. However, some infrequent symptoms also have been reported, including multiple separate synchronous lesions, hypertrichosis, hyperhidrosis, tuberous sclerosis, Williams syndrome, and so on [4, 11–18]. These less common symptoms seem to represent chance occurrences, rather than true syndrome association [4, 6, 11].

Because of the rarity of FHI, the radiologic findings of FHI have not been sufficiently investigated in the literature. On PubMed search, we found FHI-related 132 papers in which only 14 cases have detailed clinical and radiologic findings to allow an in-depth imaging analysis (Table 1) [8–10, 12–22]. Of the 16 cases of FHI, including our 2 cases, there were 9 cases undergoing X-ray imaging. X-ray imaging has little value for diagnosis of FHI because FHI lesions only demonstrate as soft tissue masses without characteristics [12, 18, 23, 24]. However, cross-sectional imaging techniques play an important role in the distinction of the lesions from the surrounding structures, and even in the determination of their histological origins. On CT and MR imaging, FHI usually presents as a soft tissue mass with the shape being oval, round flat cake, or irregular with shallow lobules [5, 10, 12, 18–21, 23–25]. According to the proportion of the different intensities similar to that of fatty tissue or muscle on CT or MR imaging, the authors classified the lesions as the balanced type or non-balanced type. In the balanced type, the proportion of the two components was basically equal [12–15, 19–22]. In the non-balanced type, however, it was not equal at all, with one of them being predominant and the other one subordinate [5, 8–10, 16–18, 23, 26]. In the first case reported by Loyer et al. who described the MR findings of FHI in 1992 [12], the lesion presented as a mass with proximate proportion of strands similar to the signal intensities of adipose or fibrous tissue. In some other cases reported in the literature, however, the lesions could also be showed as predominant intensity of fibrous tissue traversed by disperse strands of fatty component or vice versa [8–10, 16–18]. The interspersed strands usually existed in a parallel fashion, or even in a whirling appearance as in our cases [10, 12–15, 18, 24]. For some cases, the adipose or the fibrous tissue might aggregate at the local area in the lesion [19–22].

**Table 1:** The clinical and imaging characteristics of FHI

Author (No.of ref.)	Age(M)/sex	Duration (M)	Location and Extension	Accompanied Symptoms	Size (cm)	Imaging Modalities	Main Compositions and border	Specific compositions and Contrast enhancement
1. Loyer et al.(1992)^[12]^	12/F	9	Upper extremity Subcutaneous layer	Hypertrichosis Pigmentation	ND	X-ray MR	Balanced Ill-defined	
2. Song et al.(2010)^[13]^	16/M	13	Hand Subcutaneous layer and muscles	Finger contracture	ND	X-ray MR	Balanced Ill-defined	Vascularity(MR) Moderately enhanced(MR)
3. Stensby et al. (2014)^[14]^	9/M	1.5	Shoulder Subcutaneous layer	Pigmentation	4.5	MR	Balanced Ill-defined	Vascularity(MR) Moderately enhanced(MR)
4. You et al.(2018)^[15]^	13/M	9	Lumbar area Subcutaneous layer	Hypertrichosis Hyperhidrosis	4	US MR	Balanced Ill-defined	
5. Guo et al.(2011)^[19]^	120/M	120	Parapharyngeal space	Snoring	5	CT	Balanced Well-defined	Calcification(CT)
6. Chang et al.(2010)^[20]^	14/M	ND	Wrist Subcutaneous layer		3.5	X-ray MR	Balanced Well-defined	Vascularity(MR) Moderately enhanced(MR)
7. Arioni et al.(2006)^[21]^	Newborn/M	0	Knee Subcutaneous layer		9	X-ray US MR	Balanced Ill-defined	Vascularity(US) Moderately enhanced(MR)
8. Vilela et al.(2017)^[22]^	Newborn/M	0	Forearm Subcutaneous layer and muscles	Fracture of the ulna	ND	X-ray US MR	Balanced Ill-defined	Mildly enhanced(MR)
9. Choudhary et al.(2010)^[8]^	46/M	46	Foot Subcutaneous layer	Recent-onset pain	0.9	X-ray US MR	Non-balanced^a^Ill-defined	Mildly enhanced(MR)
10. Zloto et al.(2017)^[9]^	7/F	7	Orbit area Subcutaneous layer		ND	MR	Non-balanced^a^ Well-defined	Markedly enhanced(MR)
11. Ashwood et al.(2001)^[10]^	12/M	5	Wrist Subcutaneous layer		3	X-ray MR	Non-balanced^b^ Ill-defined	Vascularity(MR)
12. Yano et al.(2004)^[16]^	10/M	3	Intradural space(T10- L4)	Leg weakness	ND	CT MR	Non-balanced^a^ Well-defined	Old hematoma Moderately enhanced(MR)
13. Choi et al.(2016)^[17]^	7/M	7	Orbit area orbital fat and muscles	Hypertropia Downgaze limitation	1.3	MR	Non-balanced^a^ Ill-defined	Moderately enhanced(MR)
14. Han et al.(2009)^[18]^	48/M	18	Abdominal wall Subcutaneous layer and muscles	Tuberous sclerosis Epidermal cyst	12	CT MR	Non-balanced^a^ Ill-defined	Mildly enhanced(CT,MR)
Present case 1	566 (47 yrs)/M	505 (42 yrs)	Craniocervical area Subcutaneous layer		21.3	X-ray CT MR	Non-balanced^a^ Ill-defined	Calcifications; Vascularity Hemorrhage; Markedly enhanced
Present case 2	232 (19 yrs)/F	232 (19 yrs)	Craniocervical area Subcutaneous layer		20.2	X-ray CT MR	Non-balanced^a^ Ill-defined	Calcifications; Vascularity

Legenda:No. of ref., number of reference. M, male, F,female. Duration(M), duration of symptoms(months). ND, not described. Balanced, equal proportion of the adipose and fibrous tissue. Non-balanced, fibrous tissue predominantly^a^ or adipose tissue predominantly^b^

Of the 16 cases, 8 belonged to the balanced type, 8 to the non-balanced type. 7 cases were found being mainly composed of fibrous tissue in the non-balanced type. Relationships among the different types, the margins, and extension of the lesions were observed. There seem to be no relationships between the types of FHI and the margins of the masses, or the types and lesions’ extension. The lesions with the ill- or well-defined margins and the lesions limited to the subcutaneous layer or even extended to its underlying muscles all could be found in the cases of two types. However, of the superficially located 14 cases, two masses with the well-defined margins limited to the location of the subcutaneous layer, and twelve masses with the ill-defined margins involved not only the subcutaneous layer (*n* = 8) but also its underlying muscles(*n* = 4). Therefore, the ill-defined masses might have the potential tendency of traversing the deep fascia.

Among the different locations of FHI throughout human bodies, only 7.5% were in the craniocervical area as in our cases [4]. To our knowledge, this is the first report which delineates the radiologic findings of FHI in adults. Obviously, our cases should be in the group of non-balanced type. However, some radiologic findings of our lesions are different from most of those reported in the literature. Firstly, the lesions in our cases are larger than the lesions in the reported cases which typically are 1 to 8 cm in diameter and seldom up to 10 cm [2–4, 6, 12, 23, 25, 27]. Secondly, vascular structures could be found in the lesions in the reported cases on MR imaging [10], in our cases, however, there were more marked vascular structures that had never been seen. Thirdly, calcification was rarely found in FHI, but in our cases calcification occurred. The reason for these appearances might be the extended duration of tumor’s growth from childhood to adulthood [4, 12, 24]. For example, we could not find the chondroid component and thrombosis microscopically which usually lead to the formation of calcification, so we believe that this might be the result of long-term degenerative change. In addition, the whirling appearance in the lobules, which is relatively characteristic just as the appearance of parallel fashion, was delineated on MR imaging in our cases, especially on T_2_-weighted images [10, 12–15, 18, 24].

The differential diagnosis should be performed for distinguishing FHI from other fibroblastic/myofibroblastic and adipocytic tumours. The latter can usually be identified by the means of age at which disease occurs, involved sites, and radiologic findings. For differentiation of FHI with rich fibrous component, fat-equivalent signal across the low signal caused by collagenous component on MRI allows us to exclude tumours such as infantile fibromatosis, myofibromatosis and congenital fibrosarcomas [24–26, 28–30]. However, it might be occasionally difficult for differentiation of FHI with rich adipose tissue from the adipocytic tumours. Lipoma is a common benign tumour in which the adipose tissue is most commonly the predominant component [2, 4, 10]. However, the signals of fibrous strands and dilated vessels that do not exist in lipoma may serve as an indication for correct diagnosis [10]. Lipofibromatosis is a fibro-fatty rare benign tumor of infancy and childhood which typically involves the superficial soft tissues of the distal extremities [31–34]. On MR imaging, the tumour also demonstrates adipose signal intensity interspersed by strands of fibrous low signal intensity [31–34]. In lipoblastomas that are predominantly composed of mature fat also appears heterogeneous signal intensity much like FHI on MR imaging. Unlike FHI, lipofibromatosis and lipoblastoma with parallel and whirling appearance have not been reported [31–34].

## Conclusion

In conclusion, the radiologic appearance of our 2 cases and the reported 14 cases, especially for MR imaging can sufficiently reveal the histologic features of FHI. On MR imaging, FHI could be classified as the balanced or non-balanced type in which the presence of parallel or whirling appearance might be characteristic and can be helpful in differentiating it from the other superficial masses. The tumours’ margins could be well- or ill-defined, with the latter having the tendency of involving the underlying muscles. For adults with long history of FHI, the huge volume, calcification, and the increased vascularity might be the additional radiologic characteristics compared with infants. However, the small number of the cases listed in the report limits further disscusion of the radiologic findings of FHI, so more experience should be gotten in the future.

## Data Availability

The datasets used and/or analyzed during the current study are available from the corresponding author on reasonable request.

## Abbreviations

CT: Computed tomography
F: Female
FHI: Fibrous hamartoma of infancy
Gd-DTPA: Gadolinium- Diethylenetriaminepentaacetic acid
H&E: Hematoxylin and eosin stain
M: Male
MR: Magnetic resonance
ND: Not described
No. of ref.: Number of reference
